# Repeatability analysis of airborne electromagnetic surveys

**DOI:** 10.1186/s40929-016-0008-1

**Published:** 2016-09-09

**Authors:** Avril Hegarty, Gerry Stanley, Eugene Kashdan, Jim Hodgson, Andrew C. Parnell

**Affiliations:** 1 MACSI, University of Limerick, Limerick, Ireland; 2 UCD School of Mathematics and Statistics, University College Dublin, Dublin, Ireland; 3 Geological Survey of Ireland, Beggars Bush, Haddington Road, Dublin, Ireland; 4 School of Mathematical Sciences, Tel Aviv University, Tel Aviv-Yafo, Israel; 5 Insight: The National Centre for Data Analytics, UCD, Ireland

**Keywords:** Apparent resistivity, Functional data analysis, P-splines, TELLUS project, Weiner process, MSC 62

## Abstract

**Purpose:**

We provide methods for determining the repeatability of airborne electromagnetic surveys when conducted at different altitudes over a number of repeated flights. Our data arise from the TELLUS project carried out by the Geological Surveys of Ireland and Northern Ireland and we examine the repeatability of the apparent resistivity at different frequencies.

**Methods:**

After considering a number of issues with the data, we propose two different models from the functional data analysis literature; a Weiner process with random effects, and a penalised spline smoother.

**Results:**

Both methods arrive at the same conclusion regarding repeatability of the data; results obtained are more repeatable for flights at lower altitudes.

**Conclusions:**

The target altitude for aircraft carrying out airborne electromagnetic surveys should be as low as possible.

## Introduction

Airborne electromagnetic (AEM) surveying is a common induction technique [1–3] used to interpret subsurface geology, structure, mineralization and contamination. To conduct an AEM survey, a transmitter (coil) on the aircraft emits a sinusoidally varying current at a specific frequency. This generated magnetic field induces a secondary electric field within the ground. The receiver coil on the aircraft measures this response and the relationship between the two fields can be used to determine the apparent resistivity of the ground. Electromagnetic (EM) surveys are commonly carried out in either the frequency domain (FEM), where the effects are measured at different frequencies, or in the time domain (TEM). The study used in this paper is of the former type, the main output of which is the apparent resistivity (*R*) at different frequencies. Our main concern in this paper is the repeatability of these apparent resistivity values, especially with respect to the target altitude at which the aircraft is aiming to fly.

**Fig. 1:**
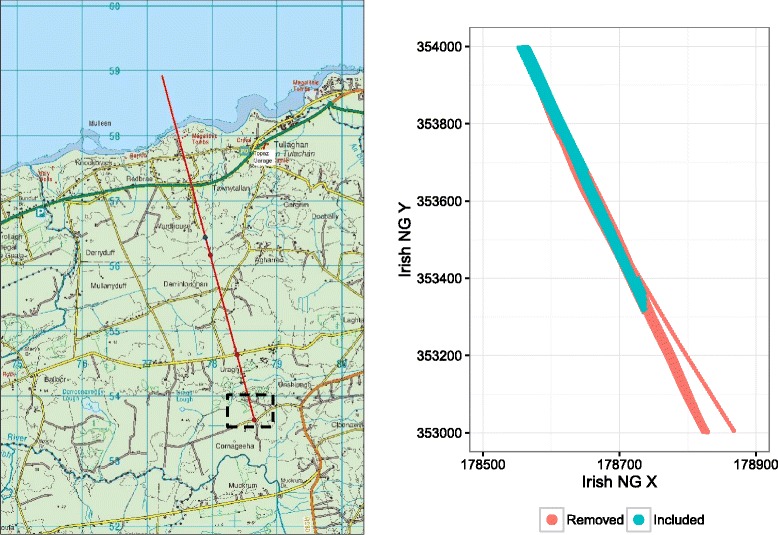
*Left* panel A map of the area covered by the testline flight. The planned flightpath is shown in *red* and the *dotted box* shows the zoomed section shown in the *right* panel. In particular note the various crossings with roads and settlements, as these are a potential cause for outlying data points. *Right* panel A zoomed-in section of the test line showing data which were obtained for individual flights. Data points marked in *red* were removed from the analysis as they were too far away from other flights to allow for repeatability testing. Irish NG X and Y refer to the Irish National Grid *x* and *y* coordinate respectively. (Copyright OSI, Licence Number: EN 0047209)

The Geological Survey of Ireland, along with the Geological Survey of Northern Ireland, carried out an FEM survey over six counties in Ireland close to the border with Northern Ireland. The project is part of a larger project funded by the EU (INTERREG IVA) and known as the tellus border project. See www.tellus.ie for reports on the airborne geophysics data processing and technical aspects of the project. Measurements were collected on two parameters at four different frequencies; the in-phase component *P* and the out-of-phase (or quadrature) component *Q*. The apparent resistivity (or its inverse, apparent conductivity) and apparent depth, which are more readily amenable to interpretation in terms of geology, can be calculated from these data using interpolation from the curves built for various frequencies and based on the homogeneous earth model [2], and after correction for many factors, including temperature and instrumental drift. See e.g. [4] for an inversion method for interpretation of FEM data. An important part of the quality-control aspect of the tellus project was to determine the effect of altitude of the aircraft on the quality and repeatability of the AEM results. To investigate this, test flights were flown along a six kilometre line at different altitudes (Fig. 1). The main objective of this paper is to analyse the data from these test flights to determine the effect of altitude on the measured data and on the repeatability of the results.

We define the term repeatability in accordance with ISO 3534-1 [5] to be:

“The closeness of agreement of independent test results obtained using the same method on identical test items in the same laboratory by the same operator using the same equipment within short intervals of time.”

We show that the test run flights are most repeatable when undertaken at the lowest feasible target altitudes.

While it is generally recognised that repeatability is an important part of AEM surveys [6] few studies have been published. Most studies that do mention repeatability assess it by repeating a test-line flight daily or on a number of occasions at a single altitude [7, 8]. For example, Green and Lane [9] analysed AEM data from a flight-line flown repeatedly at a single target altitude to monitor system performance. They described an approximate method for correcting for altitude and obtained a good measure of the repeatability after applying this correction. Foged et al. [10] investigated repeatability of airborne and ground-based TEM systems at three different altitudes (10, 20 and 30 m) and concluded that results were satisfactorily repeatable within and between altitudes, and that there was good agreement with a ground-based reference section. A more extensive study of repeatability was carried out by Huang and Cogbill [11] in which they concluded that spatially consistent flight paths are required for repeatability analysis of the EM data, and that this analysis is more meaningful if the apparent resistivity is used instead of the EM response itself. Our paper is an attempt to follow and validate this recommendation.

We analyse the tellus test-line flights to determine which altitude is the most repeatable. The target altitudes of the test flights ranged from 60 to 90 m. The lowest altitude that could safely be flown at was considered to be 60 m to avoid problems with obstacles on the ground. The stages involved in this analysis are:

1. An initial data clean-up stage to remove flights that went off course, and areas of the test-line where data were recorded over water.
2. An exploratory data analysis to determine which variables are important and to reveal any hidden structure in the data
3. Statistical models to quantify the variability between test flights that took place at the same target altitude

For each flight and for each frequency we have the aforementioned in-phase and out-of-phase components, but we focus our analysis on the apparent resistivities since the former tend to vary with altitude. This is in line with Huang and Cogbill’s conclusion [11]. To our knowledge this is the first paper to compare statistically the repeatability at different altitudes of an FEM survey.

We do not attempt to quantify any error in the apparent resistivity arising from sources other than the altitude of the flight and its position across the test line route. Furthermore, the relationship between repeatability, which we estimate via a variance computation, does not necessarily correspond to a reduction in bias. As we will show, in nearly all cases the flights that were most repeatable were those where the aircraft was aiming to fly at the lowest altitudes. This suggests that future flights to determine apparent resistivity should also be conducted at the lowest feasible altitude. However, it is possible (though unlikely) that apparent resistivity data from flights taken at the lowest altitude also contain considerable bias, and that a higher altitude may be preferable when considering both repeatability and bias simultaneously. While data for actual ground-based apparent resistivity measurements for a short 300 m section of the flight test-line was available, these data were insufficient to examine this potential bias, so we leave this as a topic for further research.

We evaluate the repeatability of the apparent resistivity via two methods from the field of functional data analysis (FDA; e.g. [12]). Since there are many different ways to analyse such data, we choose a Bayesian and a frequentist version, the former corresponding to a functional ANOVA (FANOVA) model. We evaluate the repeatability for each target altitude using a signal to noise ratio (SNR) [13] appropriate to each model. (Note that there are a number of different definitions of SNR, we use the reciprocal of the coefficient of variation). We find that both approaches reach the same conclusion; lower altitudes are more repeatable.

This paper is structured as follows. In Section “Methods” we describe the design of the study, outline our data set and perform some exploratory data analysis. The data requires careful cleaning before analysis and we document these steps here. In Section “Statistical models for measuring repeatability of apparent resistivity” we outline our two statistical approaches and detail their various advantages and shortcomings. We discuss results in Section “Results and discussion” and conclude with some ideas for further analysis in Section “Conclusions”.

## Methods

### Design of study

Test flights were flown along a 6 km test line with readings taken every 0.1 second or approximately every 6 metres. The aircraft used was a De Havilland DHC-6 twin Otter (registration number C-GSGF) for all survey work. A map of the test-line is given in the left panel of Fig. 1. One end of the test line, for approximately 1 km, was over the sea. There were 5 individual flights on different days and each of these flew up and down the line changing altitude after each turn to give 7 different altitudes, nominally at 60, 65, 70, 75, 80, 85 and 90 m. Although a target altitude was set fluctuations were unavoidable and actual altitudes were also recorded via a laser altimeter. Measurements were collected at four different frequencies: 912, 3005, 11962 and 24510 Hz referred to henceforth as 0.9, 3, 12 and 25 KHz. Negative values of apparent resistivity were ignored and replaced by interpolation from neighbouring measurement points.

### Cleaning of test flights data set

**Fig. 2:**
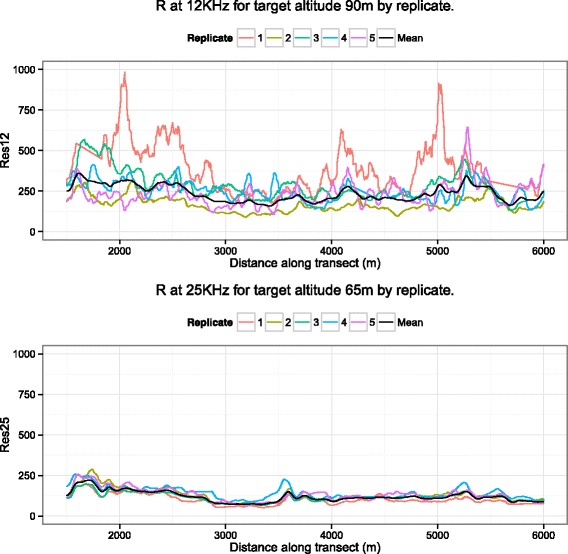
Plots of apparent resistivity values (*y*-axis) against distance along transect (*x*-axis). The individual lines represent the raw apparent resistivity values (here at frequencies 12 and 25 KHz) for each replicate. The *top* panel was obtained when flying at the target actual altitude of 90 m, whilst the *bottom* panel at 65m. The *black line* represents the modelled mean in apparent resistivity for the Weiner Process model detailed in Section “Approach 1 - Weiner process”. Straight sections of the line in Replicate 1 are due to missing data. From these two plots it appears that the lower altitude (*bottom* panel) has less variability between replicates (i.e. is more repeatable) than the higher altitude (*top* panel)

A plot of the flight paths (Fig. 1) shows that the transects were highly similar except in a few places at either end where flights occasionally went off-course. Cursory examination of the dataset reveals that the first flight (L7001) finished 500 metres further along the test line than the other 4 flights at all 7 attempted altitudes. Similarly, a large number of the measurements in the data set occur over the sea or at locations which are far from the testline where the plane may have been blown slightly off-course. We thus performed a data cleaning step where we removed all observations that lay more than 2 standard deviations from the linear regression line through the flight path as well as those over the sea. A sample of the removed data points are shown in red in Fig. 1 (right panel) which displays a zoomed-in section of the test line. As a final step, we standardised the distance variable to 10m steps so that each flight/altitude combination had approximately 450 readings for each apparent resistivity variable. The final data set we use has 25,417 observations, as opposed to the 34,898 available in the original data set. A plot of the different apparent resistivity values for two particular frequencies is shown in Fig. 2.

### Exploratory analysis

#### Weather and power line interference

We examined the weather records for the days of the flights to see if this might have had an impact on the results. However, none of the days showed particularly strong wind (the windiest day had just a moderate breeze), nor excessive rainfall in the 3 days prior to the flight, nor unusually high nor low temperatures; see Table 1. Examination of the test line showed that no major power lines crossed the flight path. However there is some suggestion in the apparent resistivity plots of anomalies along the line at about 2 and 5 km. The former of these corresponds to a main road and the latter to the built-up area of Uragh.

**Table 1:** Weather reports for the days in which the 5 different flights were taken (source: Met Éireann)

Flight	Date	Station	Rain	Max Temp	Min Temp	Mean Wind
			(mm)	°C	°C	(knots)
L7001	26/10/2011	Finner	0.3	11.0	4.5	9.8
L7025	10/12/2011	Finner	7.4	8.6	2.9	12.8
L7050	23/01/2011	Finner	2.9	8.4	3.7	10.7
L7129	08/05/2011	Finner	2.2	11.1	2.4	7.9
L7177	15/07/2011	Finner	1.9	16.2	9.6	9.1

#### Variation with altitude

One key aspect of the repeatability problem is the discrepancy between target altitude and actual altitude. During the course of the flights, the pilot was set a task of flying at a set altitude (the ‘target’ altitude), but due to weather conditions or other obstacles the actual altitude of the plane can vary widely. This causes something of a confounding problem in our approach, as it might be that poor repeatability in, e.g. apparent resistivity, is caused by the inability of the pilot to fly at that target altitude consistently, rather than because the target altitude is simply higher or lower. We thus performed an initial analysis on the data set to investigate the relationship between the variability of the actual altitude at the different target altitudes.

Two sections of the test-line were selected to examine this: Section A from 3000–3500 m where the apparent resistivity looked fairly stable, and Section B from 4800–5200 m which looked noisy (cf top panel of Fig. 2). The standard deviations of the results for these sections are given in Table 2. These results show that for the sections of the testline investigated, whilst there are differences in the standard deviations of the actual altitudes at the different target altitudes, there is no evidence of a pattern with increasing or decreasing target altitude.

**Table 2:** Comparison of standard deviations for two chosen sections of the test line data

Nominal	No. of	Mean of	Standard Deviation
Altitude	Observations	Actual Altitude	of Actual Altitude
Section A: 3000–3500 m			
90 m	415	91.1	3.76
85 m	438	84.7	6.26
80 m	418	79.4	4.06
75 m	441	74.3	5.21
70 m	397	69.7	4.35
65 m	426	65.1	5.80
60 m	408	56.9	4.41
Section B: 4800–5200 m			
90 m	341	98.5	8.91
85 m	364	86.0	10.53
80 m	327	89.3	9.79
75 m	299	74.3	9.67
70 m	331	80.3	7.75
65 m	318	65.4	8.07
60 m	327	63.9	6.50

Note that there is no clear relationship between the standard deviations and the actual altitudes. Outliers (as discussed in Section “Cleaning of test ights data set”) have been removed

### Statistical models for measuring repeatability of apparent resistivity

In this section we outline and build models for each flight’s apparent resistivity and quantify the variability between the different target altitude replicates. We explore this problem with two different approaches, both falling within the framework of Functional Data Analysis (FDA; e.g. [12]). The first approach involves fitting a single statistical model where the apparent resistivity for each frequency/altitude combination is given an overall mean modelled as a continuous time random walk (a Weiner Process, e.g. [14]) in distance, together with a random effect for replicate. Under this approach repeatability is quantified by a specific parameter in the model; the variance of the random effect. The second approach involves fitting splines individually to each frequency/altitude/replicate combination. We can subsequently calculate the variance between replicates to give an estimate of variability over the entire course of the test line. Whilst providing richer summaries, this second approach does not utilise a holistic statistical model on all of the data, and so results are more influenced by outlying values. A further contrast between the two models is in the smoothness of the stochastic process applied to the apparent resistivity. In the Weiner Process model, this is considerably rougher than the spline approach. This is a deliberate attempt to show that our conclusions are robust to the choice of statistical model.

We define, for both approaches, *y*_*ijk*_(*d*) as the natural log of apparent resistivity for frequency *i*=1,…,4, target altitude *j*=1,…,7, and replicate *k*=1,…,5 at continuous transect distance *d*. This variable forms our response. For the first approach we treat each frequency/altitude combination as independent, so for notational simplicity we write out the models as *y*_*k*_(*d*) and we ask the reader to remember that each of these models is run independently for each frequency/altitude combination. For the second approach we simplify further to write *y*(*d*) as each model is run on every frequency/altitude/replicate combination. With so many fitted models, the number of plots and results that we can display becomes cumbersome. Instead we show only those plots that we feel are of most interest, usually corresponding to those where the models fit best and worst.

#### Approach 1 - Weiner process

In Approach 1 we model the response as coming from an underlying mean process which we set as a Weiner Process to capture the variability in apparent resistivity along the transect. We further include a random effect to account for the discrepancy between replicates at the same altitude. The model formulation is as follows:

$$y_{k}(d) = \mu(d) + b_{k} + \epsilon_{k}(d) $$

where $\mu (d) \sim N(\mu (d-\Delta d),\sigma ^{2}_{\mu } |\Delta d|)$ is a Weiner Process with *Δ* *d* a small change in *d* and $\sigma ^{2}_{\mu }$ the Weiner process variance. $b_{k} \sim N(0,{\sigma _{b}^{2}})$ is the additive random effect of each replicate, and *ε*_*k*_(*d*)∼*N*(0,*σ* ^2^) is a model error term. The key parameter here is *σ*_*b*_; our replicate variability for that altitude/frequency combination. We determine the performance of the model by calculating the Signal to Noise Ratio (SNR) [13] by the formula:

$$ \text{SNR}(d) = \frac{{\mu}(d)}{\sigma_{b}+\sigma} $$

Different frequencies have different SNR ratios and different penetration depths; higher frequencies have higher SNRs than lower frequencies. However, high frequency signals decay very fast and the penetration of lower frequencies is deeper. A good model will have a higher SNR, as both the within-replicate variability term *σ* and the between-replicate variability *σ*_*b*_ will be small in comparison to the level of signal as represented by *μ*. When calculating the final SNR, we average over distance via $\frac {1}{N} \sum _{i=1}^{N} \text {SNR}(d_{i})$, where *N* is the number of unique distances. We thus get a single estimate of SNR from the model, which allows us to compare different target altitudes via boxplots (see “Results” Section).

We fitted the Wiener Process model using the Bayesian Hamiltonian Monte Carlo package stan [15] using half-Cauchy weakly informative priors on the standard deviation terms *σ*_*μ*_,*σ*_*b*_ and *σ*. We ran the model for 1000 iterations on 4 chains, for each of the target altitudes at each of the 4 different frequency values (0.9, 3, 12 and 25 KHz), totalling 28 model runs. On a 3.4 GHz Core i7 Processor with 16 Gb of RAM the computing took about 12 h. The main advantage of using Hamiltonian Monte Carlo is that far fewer iterations are required as it more efficiently explores the posterior parameter space [16]. We remove 200 iterations for burn-in and checked for convergence using the standard Brooks-Gelman-Rubin statistic [17, 18]. A more complete joint model incorporating all frequency/altitude/replicate combinations was attempted but found to be too computationally expensive.

#### Approach 2 - splines

For our second approach we use Penalised Splines (P-Splines; [19]) on each of the altitude/replicate/frequency combinations. The model is thus:

$$y(d) = B(d)^{T} \beta + \epsilon(d) $$

where *B*(*d*) is a vector of *K* cubic B-spline basis functions for distance *d*,*β* is a vector of weights for each B-spline, estimated as part of the model, and *ε*(*d*)∼*N*(0,*σ* ^2^) is a model error term as in Approach 1. As standard, we penalise the second differences of *β* so that the spline remains smooth. The model is thus fitted by including a penalty term where the degree of smoothing is controlled by another parameter *λ*. We fit the P-spline model by minimising the objective function:

$$\sum_{d} \left(y(d) - B(d)^{T} \beta \right)^{2} + \lambda \left(\Delta^{2} \beta\right)^{T}\left(\Delta^{2} \beta\right) $$

where *Δ* indicates a first difference. We estimate *λ* via cross validation.

We fit the above model using the frequentist smooth.spline function in R [20] with *K*≈100 basis functions (the exact number is set for each run by the function according to the response variability). As stated above, we run the model for each of the 4 frequencies (900 Hz, 3, 12 and 25 KHz) at each of the 7 altitudes and each of the 5 replicates, a total of 140 runs. We estimate *λ* via 10-fold generalised cross validation where the optimisation criteria is the root mean square error (RMSE). The smoothed functions are derived and the average and standard deviation functions were calculated across the different distance values. Since the model fitting in this approach is a deterministic procedure the computing time required is a matter of seconds.

For this approach we compute the SNR as:

$$\text{SNR}(d) = \frac{B(d)^{T} \hat{\beta}}{\hat{\sigma}_{b}(d)} $$

where $\hat {\beta }$ is the estimated spline weight and $\hat {\sigma }_{b}(d)$ is the estimated standard deviation across replicates at distance *d* between the 5 spline fits at that altitude. Note that under this approach we have no estimate of the within altitude variability so the uncertainty in the SNR is likely to be different from that of the Weiner Process. We thus use the SNR to compare the performance of the models between target altitudes. We do not use the two versions of the SNR to compare between models.

## Results and discussion

We fitted the Weiner Process model to the apparent resistivities for each frequency/altitude combination to get a mean apparent resistivity over the sets of five replications. See Fig. 2 for two data cases chosen to showcase a situation of low repeatability (12 KHz at target altitude 90 m), and high repeatability (25 KHz at target altitude 65 m). The estimated posterior mean apparent resistivity *μ*(*d*) is shown as a solid black line and the raw data as coloured lines.

**Fig. 3:**
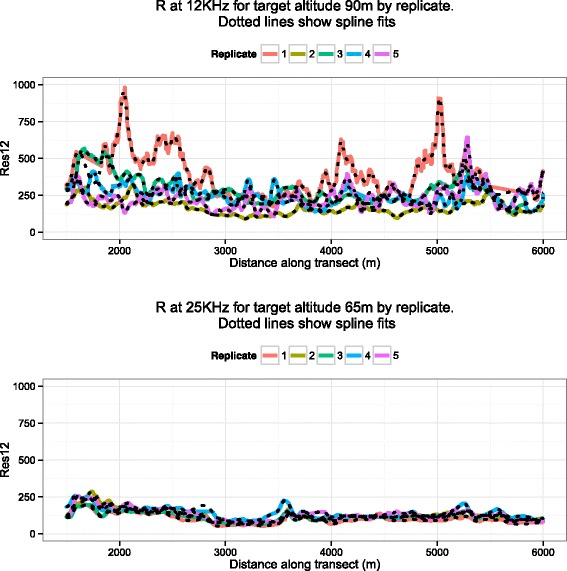
Plots of apparent resistivity values (*y*-axis) against distance along transect (*x*-axis). The data for both panels are the same as Fig. 2. The *black dotted lines* indicate the spline fits for each of the replicates and the *solid coloured lines* represent the raw apparent resistivity values. As before, it appears that the lower altitude mean (*bottom* panel) has less variability around it than the higher altitude (*top* panel). For the spline model we can evaluate the repeatability by comparing SNRs (see Fig. 5). Note that the dots diverge from the coloured lines where data are missing

The spline model was fitted to each frequency/altitude/replication and the results are shown in Fig. 3 for the same data cases as for the Weiner model in Fig. 2. The dotted lines show the fitted values for each of the replicates and as in Fig. 2 the raw data is shown as coloured lines. Note that this second approach fits the data far better by fitting to each replicate, but is likely to suffer from over-fitting due to the flexibility of the spline model.

**Fig. 4:**
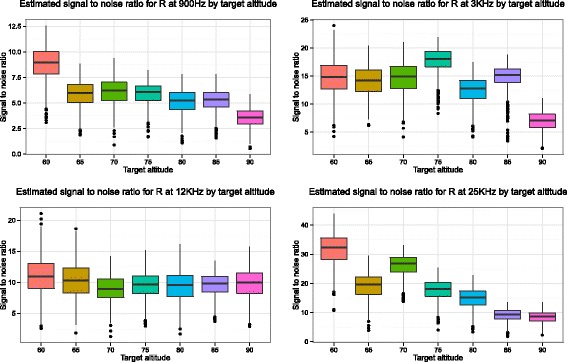
Boxplots of the estimated signal to noise ratio (*y*-axis) versus the target altitude (*x*-axis) for the four different frequencies based on apparent resistivity (*R*) values. These are created from the output of the Weiner Process model detailed in Section (“Approach 1 - Weiner process”). Higher values indicate that there is better preservation of signal. For both the 900 Hz frequency and 25 KHz frequency flying at lower altitudes seem strongly desirable. This is less clear however at frequencies 3 and 12 KHz

**Fig. 5:**
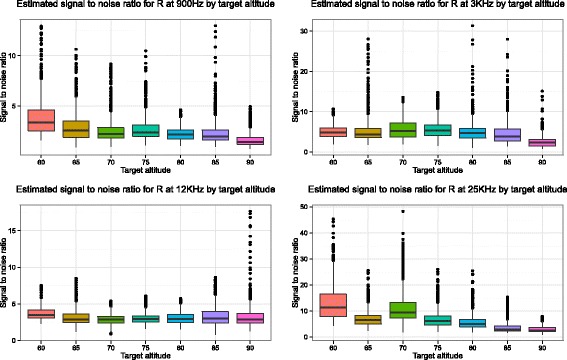
Boxplots of the signal to noise ratio for the spline models by target altitude for each frequency. Higher values indicate more repeatable results. As for the Weiner Process model, it appears that lower target altitudes give higher signal-to-noise ratios. Note, however, the larger number of outliers here, due to the greater flexibility in the spline model

The repeatability of the models is assessed by the signal-to-noise ratios, which is computed from the output of the two models and is represented using boxplots in Figs. 4 and 5. Higher values indicate that there is better preservation of signal and therefore better repeatability. We can clearly see that the lower altitudes have higher SNRs, especially at 900 Hz and 25 KHz. The SNRs for the spline models are more variable due to the closer fit of the model to the data. However, from the plots taken together for both models we can draw a clear conclusion; lower flights are more repeatable. The improved repeatability of lower altitude flights is shown in both the Weiner Process and Spline models despite their different smoothness characteristics (as detailed in Section “Statistical models for measuring repeatability of apparent resistivity”). Repeating the analysis for the in-phase (*P*) and quadrature components (*Q*) yields the same conclusions (for brevity not shown).

**Fig. 6:**
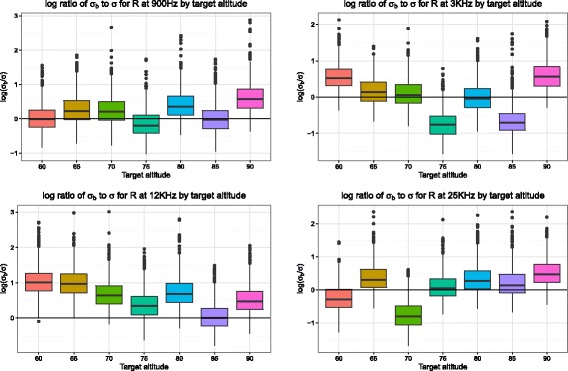
Boxplots of the log ratio of *σ*_*b*_/*σ* (y-axis) versus the target altitude (x-axis) for the four different frequencies based on apparent resistivity (R) values. Higher values (above zero) indicate that the variability between replicates (*σ*_*b*_) was greater than that of the internal variability (*σ*). The majority of values appear to be concentrated around zero, indicating approximate equality. However, at 12 Khz the variability between runs appears in general higher than that of the internal variability

The identifiability of the Wiener models is shown in Fig. 6 as the ratio of the between-replicate variability *σ*_*b*_ to the within-replicate variability *σ* based on apparent resistivity (R) values. These are created from the output of the Weiner Process model. Higher values (above zero) indicate that the variability between runs (*σ*_*b*_) was greater than that of the internal variability (*σ*). The majority of values appear to be concentrated around zero, indicating approximate equality. However, at 12 Khz the variability between runs appears in general higher than that of the internal variability.

## Conclusions

We applied two models using very different approaches, one Bayesian (Weiner process) and one frequentist (Spline model). Boxplots of the results show that for both models the conclusion regarding repeatability of apparent resistivity, over the range of altitudes 60–90 m, is that lower altitudes give more repeatable results than higher altitudes. Note however that due to lack of data, the conclusion regarding repeatability ignores any possible bias in the apparent resistivity at the different altitudes arising from any source i.e. it is possible that the lower altitude measurements contain more bias than the higher altitudes.

Several opportunities present themselves for future research. First, it would be desirable to quantify both repeatability and bias in the apparent resistivity measurements. To do this we would need ground-based measurements across a long segment of the test line. A second extension would be to run a richer cross validation experiment to determine which amongst a larger family of statistical models fit the data (and so quantify replication) best. A final possible extension would be to include a richer Weiner Process or Spline model that treats all of the data simultaneously. We leave such an extension to another paper.

## Abbreviations

AEM: Airborne electromagnetic
EM: Electromagnetic
FEM: Frequency (domain) electromagnetic
SNR: Signal to noise ratio
TEM: Time (domain) electromagnetic
